# Comparison of Sandstone Damage Measurements Based on Non-Destructive Testing

**DOI:** 10.3390/ma13225154

**Published:** 2020-11-16

**Authors:** Duohao Yin, Qianjun Xu

**Affiliations:** State Key Laboratory of Hydroscience and Engineering, Tsinghua University, Beijing 100084, China; ydh16@mails.tsinghua.edu.cn

**Keywords:** non-destructive testing, P-wave velocity, amplitude attenuation, resistivity, CT scan, sandstone, damage variable

## Abstract

Non-destructive testing (NDT) methods are an important means to detect and assess rock damage. To better understand the accuracy of NDT methods for measuring damage in sandstone, this study compared three NDT methods, including ultrasonic testing, electrical impedance spectroscopy (EIS) testing, computed tomography (CT) scan testing, and a destructive test method, elastic modulus testing. Sandstone specimens were subjected to different levels of damage through cyclic loading and different damage variables derived from five different measured parameters—longitudinal wave (P-wave) velocity, first wave amplitude attenuation, resistivity, effective bearing area and the elastic modulus—were compared. The results show that the NDT methods all reflect the damage levels for sandstone accurately. The damage variable derived from the P-wave velocity is more consistent with the other damage variables, and the amplitude attenuation is more sensitive to damage. The damage variable derived from the effective bearing area is smaller than that derived from the other NDT measurement parameters. Resistivity provides a more stable measure of damage, and damage derived from the acoustic parameters is less stable. By developing P-wave velocity-to-resistivity models based on theoretical and empirical relationships, it was found that differences between these two damage parameters can be explained by differences between the mechanisms through which they respond to porosity, since the resistivity reflect pore structure, while the P-wave velocity reflects the extent of the continuous medium within the sandstone.

## 1. Introduction

Sandstone is a natural building material and also a common lithology found in the rock surrounding underground engineering sites such as tunnels and underground powerhouses [1]. Sandstone is generally characterized by low strength and high permeability, making it a weak link in the surrounding rock [2]. In underground rock mass engineering, various disturbances will cause damage to the sandstone. Accumulation of sufficient damage will then lead to rock mass failure and affect the stability of the surrounding rock during construction and operation. Rock damage can be measured using a damage test, which provides parameters from which damage variables can be calculated that reflect the damage and can be used to study its evolution. Mechanical parameters such as the elastic modulus and plastic dissipation energy are commonly used for defining damage variables. In addition, damage variables can also be derived from non-destructive testing (NDT) parameters, such as the ultrasonic velocity, wave amplitude attenuation, resistivity and effective bearing area based on computed tomography (CT) scans [3]. These NDT parameters can be obtained faster and more easily than elastic modulus. However, due to the limitations of the measurement methods and their accuracy, these parameters have not been widely used in rock damage mechanics research [4]. This motivates the comparative study of non-destructive measurement parameters that can be used to define the damage variable.

Acoustic testing, including ultrasonic and acoustic emissions testing, are common non-destructive testing (NDT) methods for rocks [5]. Alemu found through testing that the strength of the rock is positively correlated with the wave velocity. When cracks appear in the rock, the strain increases and the wave velocity decreases until the rock is considered to be failed [6]. Heap found that an increase in the peak stress used for the cyclic loading significantly reduced the dynamic elastic modulus of the basalt obtained from ultrasonic tests [7]. As an ultrasonic wave propagates inside a rock, the wave amplitude attenuates at any internal defects. The amplitude attenuation of waves travelling through rock is very sensitive to rock fracture, making it useful for the definition of damage variables [8]. Yim believed the attenuation of ultrasonic waves in rocks is closely related to the properties of internal defects and the porosity of the rock [9]. The simplest measure of ultrasonic wave attenuation is the difference in amplitude between the transmitted wave and the first received wave. Muller suggested that the first wave amplitude attenuation can be taken to reflect rock damage, but the measurement of this parameter is challenging [10].

In addition to ultrasonic parameters, electrical resistivity can be used to define rock damage. Ranade tested the response of resistivity to defects in cement under tension and used resistivity to study the damage evolution [11]. Kahraman found that resistivity is more sensitive to porosity than acoustic velocities, but the direct current (DC) electrical resistivity measurement limits the accuracy of rock resistivity [12]. At present, electrical impedance spectroscopy (EIS) based on alternating current provides the most accurate measurements of rock resistivity. Zisser used the EIS test to study the anisotropy of permeability in dense sandstone [13]. Our previous paper found that the resistivity obtained from the EIS has a high accuracy and can be used to define damage variables in sandstone [14].

From a microstructural perspective, rock damage reflects a change in the effective bearing area. However, current rock measurement techniques struggle to accurately capture the effective bearing area and it can only be measured using expensive meso-testing methods such as CT scanning [15]. Yin observed the damage of granite after ultrasound-assisted rock breaking using CT scan tests [16]. Landis established the damage degree of rock by counting instances of defects extracted from CT images [17].

The aforementioned NDT methods all have their unique advantages. Longitudinal wave (P-wave) velocity and CT scan testing are commonly used in rock damage studies, while resistivity and wave amplitude attenuation are less frequently adopted due to limitations in measurement method and accuracy. Shah compared P-wave velocity and amplitude attenuation that are measured in ultrasonic tests and concluded that they can both be used to reflect rock damage [18]. Yang used two loading methods to cause damage to sandstone, and then defined damage variables using the P-wave velocity and compared them to damage variables that were defined using the elastic modulus [19]. However, systematic comparative studies among different NDT methods are still lacking.

To verify the accuracy of damage variables derived from NDT parameters, this study compared damage variables derived from P-wave velocity, amplitude attenuation, resistivity and the effective bearing area calculated from a CT scan with the damage variables obtained from elastic modulus for sandstone. The comparison considered four areas of difference: differences between the parameters used to define the damage variables; consistency between the different damage variables and the stability of each of the damage tests; differences between microscopic and macroscopic measures of the damage; and differences between the mechanisms that influence measurements made using the acoustic and electrical tests.

## 2. Materials and Methods

### 2.1. Sandstone Specimens and Pretreatment

**Figure 1:**
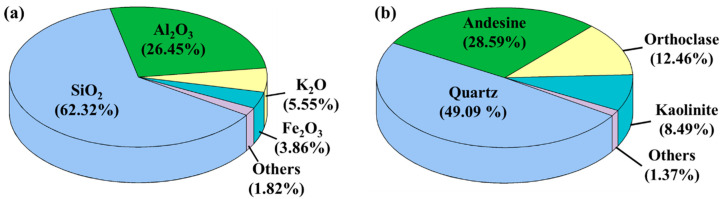
(**a**) Major oxides and (**b**) major minerals composition of rock (wt. %).

Following ISRM standards and the laboratory’s measuring instrument conditions, a representative homogeneous and coarse-grained arkose sandstone core was selected for this study and processed into twelve 75 mm long, cylindrical specimens with diameters of 37.5 mm. The arkose sandstone was taken from Hunan Province, China, and the specimens were drilled from an adjacent location on the same rock block. The two end faces of the specimen were ground to make them parallel to each other and perpendicular to the axis of the cylinder. The physical and mechanical properties of the specimens were measured and the porosity was found to be 14.9%, the dry density was 2313.08 kg/m^3^, and the strength was 18.59 MPa. The oxides of the sandstone specimens were found using X-ray fluorescence spectroscopy (XRF), and the minerals composition was obtained by X-ray diffraction (XRD) testing. As shown in Figure 1b, the major mineral compositions of the specimens are quartz and feldspar, and the major clay composition is kaolinite, accounting for 8.49%.

**Figure 2:**
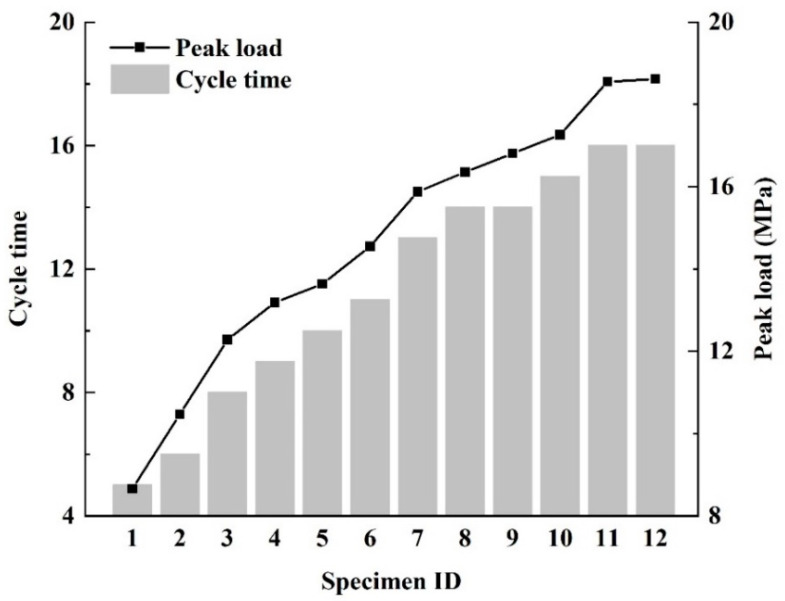
Cyclic loading path for each sandstone specimen.

Prior to the test, the specimens were placed in a drying oven, heated at 105 °C for 12 h and then cooled naturally, after which the specimens were saturated in a vacuum saturator for 22 h. Uniaxial cyclic loads with different loading paths were applied to each of the twelve specimens so that they were each subjected to different damage, as shown in Figure 2.

**Figure 3:**
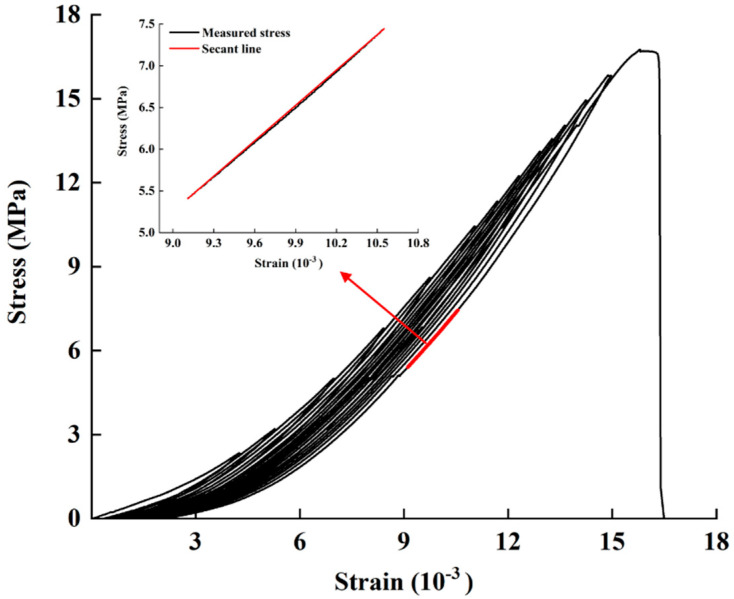
Stress-strain curve measured for the specimen No. 11 during cyclic loading.

As shown in Figure 3, stress and strain were measured simultaneously for the specimens during the uniaxial cyclic loading. The portion of the unloading section that accounts for 30%–40% of the strength is assumed to be the straight line section of the stress-strain curve, marked in red in Figure 3, and its secant elastic modulus is taken to be the elastic modulus for the sandstone specimen. The elastic modulus for the first loading cycle in which the peak load exceeds 40% of the strength is taken as the elastic modulus of the specimen before damage, and the elastic modulus of the last loading cycle is taken as the elastic modulus after damage. The equivalent strain assumption allows these to be used to obtain the sandstone damage variable for sandstone derived from the elastic modulus [20]:

(1) $$D=1-\frac{E_{D}}{E_{0}}$$

where *D* is the damage variable; *E*_0_ and *E_D_* are the elastic modulus (GPa) for the initial state and for the damaged state, respectively.

### 2.2. Non-Destructive Testing

#### 2.2.1. Ultrasonic Test

**Figure 4:**
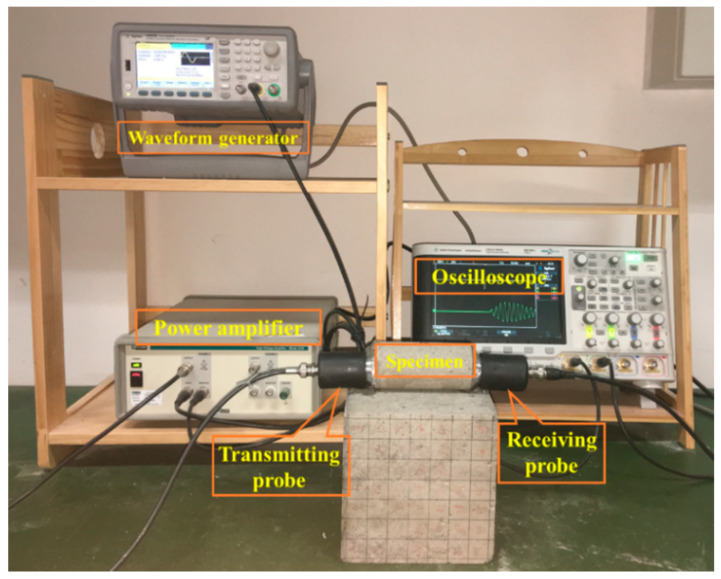
Ultrasonic testing setup.

Ultrasonic parameters are largely determined by the internal structure of the rock and reflect the damage properties of the rock. A 33522A ultrasonic tester (Agilent, Santa Rosa, CA, USA) was used to measure the P-wave velocity and first wave amplitude attenuation for the sandstone specimens in the initial state, and as the damage state evolved following the cyclic loading, as shown in Figure 4. After pretreatment, the saturated sandstone specimens were tested with an ultrasonic wave frequency of 50 kHz. The acoustic test was calibrated using a standard aluminum block and an appropriate amount of vaseline was smeared between the ultrasound probe and the specimens to ensure close contact. The specimens were tested in a stress-free state at room temperature, and the ultrasonic probes were fixed to both ends of the specimens.

**Figure 5:**
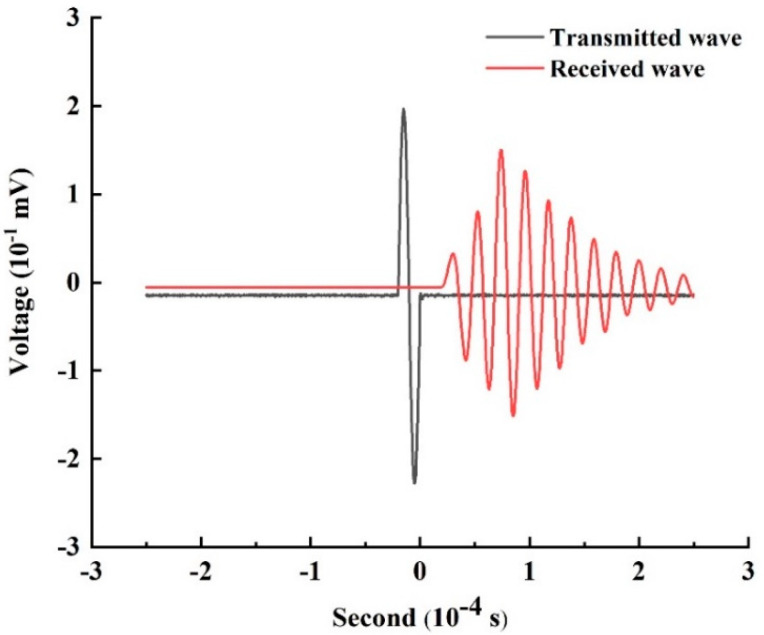
Transmitted and received waves.

The transmitted and received waves can be captured using an oscilloscope as shown in Figure 5, where the voltage represents the wave amplitude. The P-wave velocity and first wave amplitude attenuation can be calculated for the specimens from the captured waveform. The P-wave velocity is equal to the length of the specimen divided by the time interval between the first maximum in the transmitted wave spectrum and the first maximum in the received wave spectrum. The first wave amplitude attenuation is the ratio of the amplitude of the first received wave to the amplitude of the transmitted wave. The damage variable was calculated from the measured parameters using the following equations [21]:

(2) $$D=1-\frac{{V_{D}}^{2}}{{V_{0}}^{2}}$$

(3) $$D=1-\frac{F_{D}}{F_{0}}$$

where *D* is the damage variable; *V*_0_ and *V_D_* are the P-wave velocity (m/s) for the initial state and for the evolving damaged state, respectively; *F*_0_ and *F_D_* are the first wave amplitude attenuation for the initial state and for the evolving damaged state, respectively.

#### 2.2.2. EIS Testing

**Figure 6:**
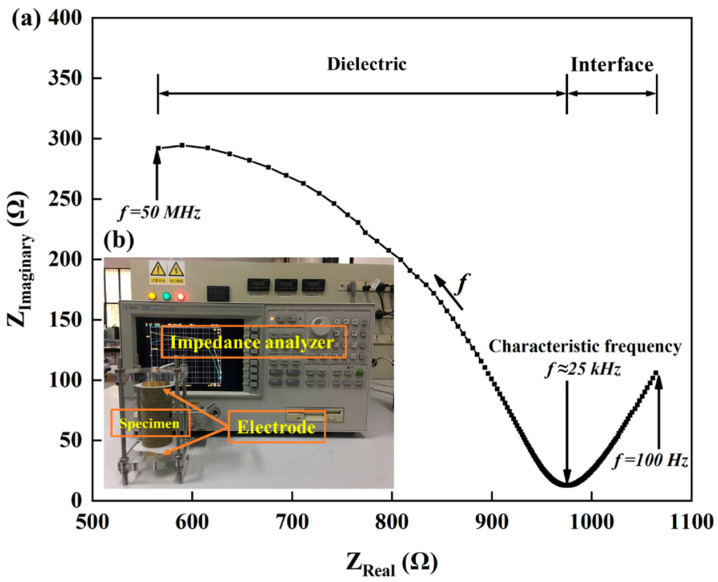
(**a**) Nyquist diagram and (**b**) EIS testing.

The sandstone specimens were saturated with sodium chloride solution and subjected to EIS testing before and after cyclic loading using an Agilent 4294A precision impedance analyzer, as shown in Figure 6b. The modulus and phase angle of the electrical impedance were measured for frequencies from 100 Hz to 50 MHz. Each specimen was tested three times and the mean of these was recorded. Please refer to our previous paper for a detailed description of the test [14].

As shown in Figure 6a, The EIS can be visualized by displaying the modulus and phase angle of the impedance as a Nyquist plot on the complex plane [22]. In the Nyquist diagram, frequency increases from right to left and the EIS is composed of arcs, corresponding to high-frequency impedance, and straight lines, corresponding to low-frequency impedance. For the saturated sandstone specimens, the high-frequency part of the diagram reflects the electrical characteristics of the combined system of rock and pore fluid, and the low-frequency part reflects differences between the electrical properties of the specimen and of the electrode. The frequency corresponding to the intersection of the high- and low-frequency parts of the diagram (around 25 kHz) is called the characteristic frequency and the corresponding resistance is called the characteristic resistance [23,24].

The characteristic resistance was converted to resistivity using the law of resistance, and the difference between the resistivity for the initial and damaged states was used as a measure of the damage of the sandstone specimens. Since current intensity obeys the equivalence assumption in damage mechanics, the damage variable derived from resistivity can be expressed using the following equation [11]:

(4) $$D=1-\frac{\rho_{D}}{\rho_{0}}$$

where *D* is the damage variable; *ρ*_0_ and *ρ_D_* (Ω.m) are the resistivity for the initial state and after damage, respectively.

#### 2.2.3. CT Scan Test

Meso-measurement methods such as CT scanning provide an important means of examining rock damage. During the damage process, new pores and cracks are formed in the rock and existing, previously isolated, pore spaces can become connected via the newly created cracks. Materials with different densities attenuate X-ray energy differently and so CT scanning can distinguish pores in the rock [25].

**Figure 7:**
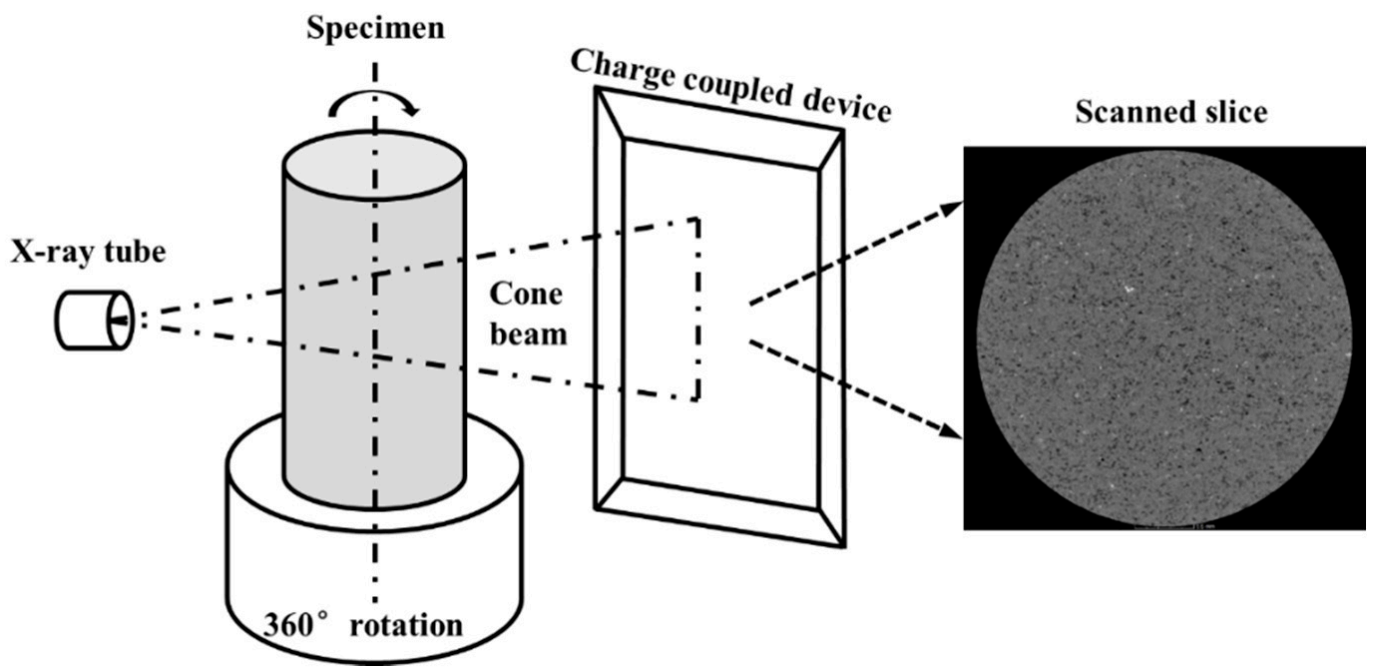
Schematic illustration of the CT system.

Three representative sandstone specimens (No. 6–8) were selected for CT scan testing before and after damage using a d2 industrial CT system (Diondo, Hattingen, Germany). Each specimen was subjected to a different cyclic load, and the ratio of peak load to strength were 78%, 85% and 88% for specimens 6, 7 and 8, respectively. A schematic illustration of the CT system is shown in Figure 7. Excluding the two ends, the scanning height is 70 mm, and a series of scanned cross-sections of the sandstone specimens were obtained at intervals of 0.5 mm.

CT images that show the pore structure before and after damage can therefore be used to characterize the damage of sandstone specimens. For each specimen, the section for which the pore area covered the greatest proportion of the section after damage was selected first, and then the corresponding section from the CT scan image before damage was selected. The effective bearing area was calculated for each specimen by removing the pore area from the total area before and after the damage separately, allowing the damage variable to be calculated as follows [26]:

(5) $$D=1-\frac{A_{Pore}}{A_{0}}$$

where *D* is the damage variable; *A_Pore_* and *A*_0_ are the pore area and the total area (m^2^) of the scanned slices, respectively.

## 3. Results

### 3.1. NDT Test Results

**Figure 8:**
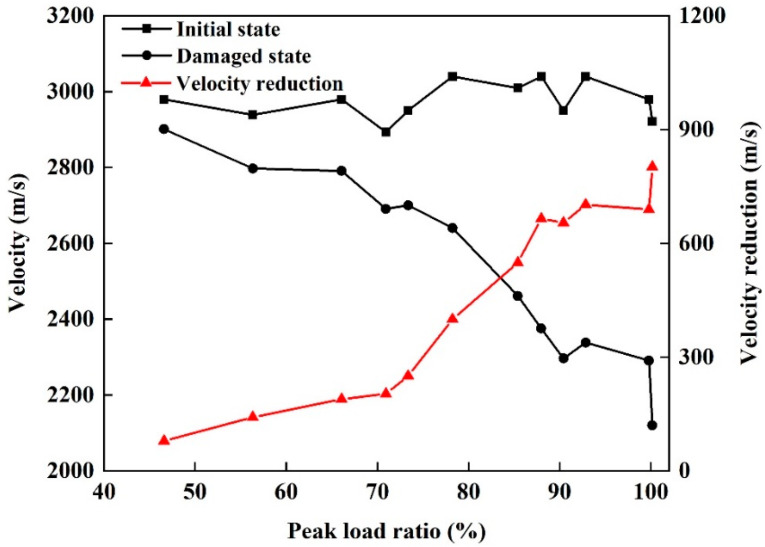
Wave velocities for sandstone specimens before and after damage.

An ultrasonic test, an EIS test, and a CT scan test were performed for each specimen before and after damage respectively, then the corresponding resulting parameters were obtained as follows. The P-wave velocities for the twelve sandstone specimens before and after damage under different peak loads and loading cycles are shown in Figure 8. For the initial state, the distribution of P-wave velocities measured for the sandstone specimens is concentrated at around 3000 m/s, with a range of 2900–3100 m/s. For a single specimen, damage leads to a decrease in the P-wave velocity. However, the reduction in wave velocity varies between specimens with different levels of damage. The wave velocities for the sandstone specimens in a damaged state range from 2900 to 2100 m/s, and the decrease, relative to the velocity for the initial state, becomes increasingly significant as the damage increases. In particular, when cracks appeared in the sandstone, the wave velocity decreased by more than 30%, compared with the velocity before damage.

**Figure 9:**
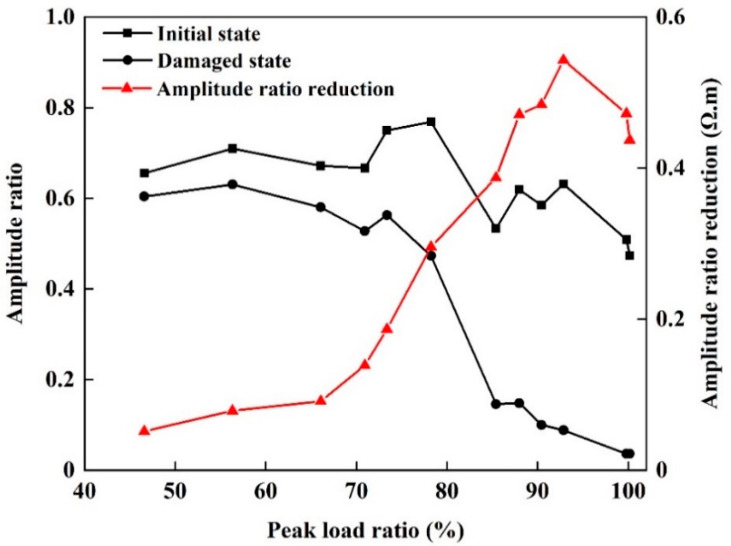
First wave amplitude attenuation for sandstone specimens before and after damage.

The measured wave amplitude attenuation increased after damage for all twelve sandstone specimens, as shown in Figure 9. The distribution of wave amplitude attenuations for specimens before damage is narrow and is centered on 0.6. When the damage is small, the difference between the wave amplitude attenuation before and after damage is also small. Similar to the P-wave velocity, as the degree of damage increases, the decrease in the wave amplitude ratio, relative to the initial state, becomes greater. When the specimen cracks after cyclic loading, the wave amplitude ratio falls below 0.2. This indicates that the first wave amplitude attenuation is more sensitive to damage than the P-wave velocity when the level of damage approaches failure.

**Figure 10:**
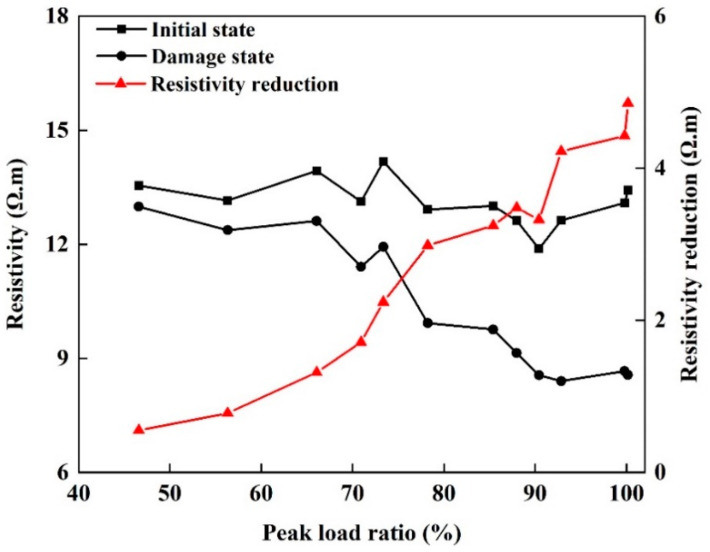
Sandstone resistivity before and after damage under different loading conditions.

Figure 10 shows the resistivity of the twelve sandstone specimens before and after damage under different peak loads and loading cycles. As the loading cycle and peak load increased, the damage accumulated and the resistivity tended to decrease. However, the rate of decrease in resistivity was not uniform. As the damage increased, the rate of decrease in resistivity also increased significantly.

In the initial state, the resistivity of sandstone was concentrated around 13 Ω.m, with individual values in the range 12–14 Ω.m. With increasing peak load and loading cycles, the resistivity of sandstone in the damage state showed varying rates of decline and was distributed in the range 8.5–13 Ω.m. When the ratio of peak load to strength was small (after few loading cycles), the resistivity was not much lower than that before damage. As the ratio of peak load to strength and the number of loading cycles increased, the decrease in resistivity became increasingly obvious. Once penetrating cracks had developed, the resistivity of the sandstone specimen dropped sharply (by about 40%) compared to that before the damage.

**Figure 11:**
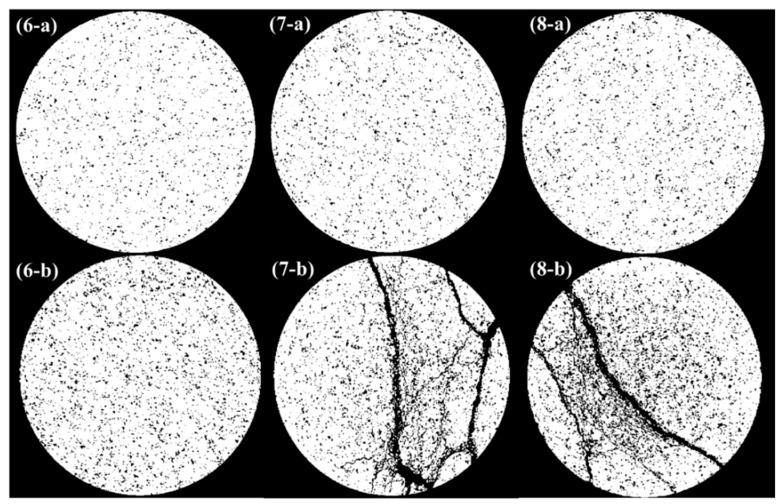
CT scan slice image after being segmented (6–8 represent the specimen ID, while a and b indicate the initial state and damage state respectively).

In CT scan slice images, the different gray levels of the pixels represent different densities of substance. The pores have relatively smaller grayscale values, corresponding to darker pixels, while the matrix has relatively larger grayscale values, corresponding to lighter pixels. Therefore, the pores within the specimen can be identified by selecting a specific threshold value of grayscale. Due to the different cyclic loads applied to specimens No. 6–8, the specimens presented significantly different pore structures after damage. Threshold segmentation was performed on the scanned slices and the pore condition before and after damage was counted separately. Figure 11 shows that the pores and cracks in the damaged specimens increase significantly as the degree of damage increases.

The change in porosity at the same section before and after damage was obtained by counting the pore area in the grayscale image. In the initial state, the porosity in the section obtained from CT scan is around 12%, with a small dispersion. Specimen No. 6 was not broken after damage, so the change in porosity was relatively small, increasing from 11.8% to 15.5%. The remaining two specimens were both fractured after damage, with a large number of new pores distributed along the cracks and a significant change in porosity. The porosity of specimen No. 7 increased from 12.76% to 19.07% and that of specimen No. 8 increased from 12.74% to 19.67%.

### 3.2. Damage Variables for Sandstone

The above results show that the four NDT parameters: P-wave velocity, first wave amplitude attenuation, resistivity, and the effective bearing area obtained from the CT scan test can all reflect damage for sandstone. The damage variables obtained from ultrasonic testing are calculated from the P-wave velocity and first wave amplitude attenuation before and after damage. The resistivity obtained from the EIS test, the effective bearing area obtained from the CT scan test, and the elastic modulus obtained from the cycle loading test can also be used to derive damage variables for all twelve sandstone specimens.

**Figure 12:**
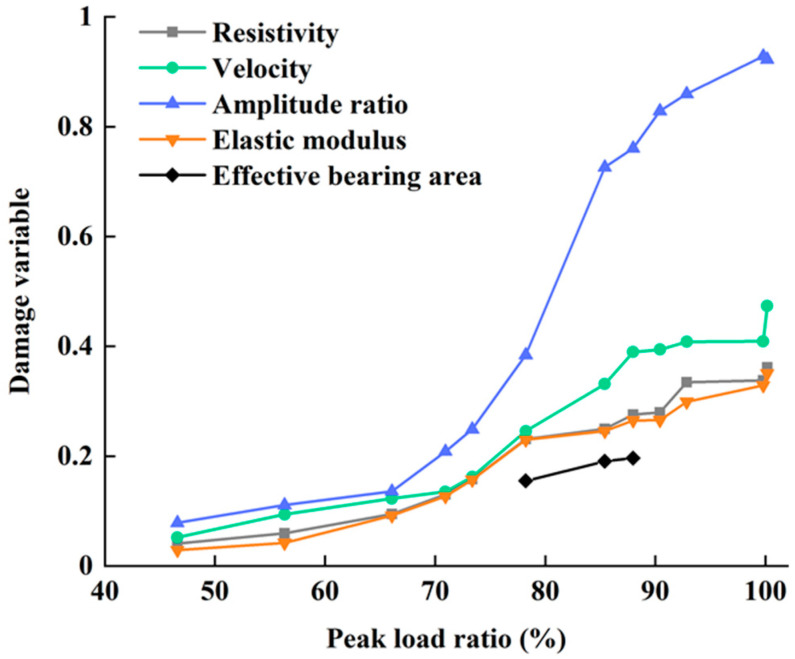
Damage variables derived from different measurement parameters. Note that three specimens were tested using a CT scan.

Figure 12 shows the differences between the damage variables for sandstone that were derived from the five different measured parameters. All five damage variables increased monotonically as the peak load and cycle times increased. As the values of the damage variables derived from these parameters differ significantly, the consistency between the responses of the different parameters to damage was further discussed. The P-wave velocity is most consistent with the elastic modulus, with a correlation coefficient of 0.991, followed by the resistivity and wave amplitude attenuation, both with a correlation coefficient of 0.885. Among the NDT parameters, the correlation coefficient between P-wave velocity and resistivity was the highest at 0.991 and that between P-wave velocity and amplitude attenuation was 0.958. The correlation coefficient between resistivity and amplitude attenuation is relatively low at 0.939. The high correlation coefficients show that the four macroscopic damage parameters, P-wave velocity, first wave amplitude attenuation, resistivity and elastic modulus, responded consistently to damage.

The damage variables derived from the P-wave velocity and wave amplitude attenuation that were measured in the ultrasonic test were greater than the damage variables that were derived from the other three measurement parameters. The damage variables derived from the first wave amplitude attenuation were greater than those derived from the P-wave velocity. This shows that ultrasonic testing is more sensitive to damage than other tests for sandstone, and that wave amplitude attenuation is the most sensitive parameter to the damage. The measured resistivity and acoustic parameters are NDT parameters. Although the damage variable derived from resistivity is smaller than those derived from the ultrasonic test measurements, it is slightly greater than the damage variable that is derived from the elastic modulus. This suggests that acoustic and electrical damage measurement parameters, in addition to being non-destructive, are superior to the elastic modulus tests in terms of their sensitivity to damage.

CT scan testing allows us to find the effective bearing area for a sandstone specimen, which can be used to define microscopic damage variables. Figure 12 shows the damage variables derived from the effective bearing area for specimens No. 6–8 and compares these with the macroscopic damage variables. The damage variables derived from the CT scan test increase with the degree of damage, in agreement with the macroscopic damage variables. However, the CT scan test is limited by resolution and is insensitive to smaller pores, with the result that the damage variable derived from the effective bearing area is smaller than the damage variables derived from the other damage parameters.

## 4. Discussion

### 4.1. Stability of Damage Measurements

**Figure 13:**
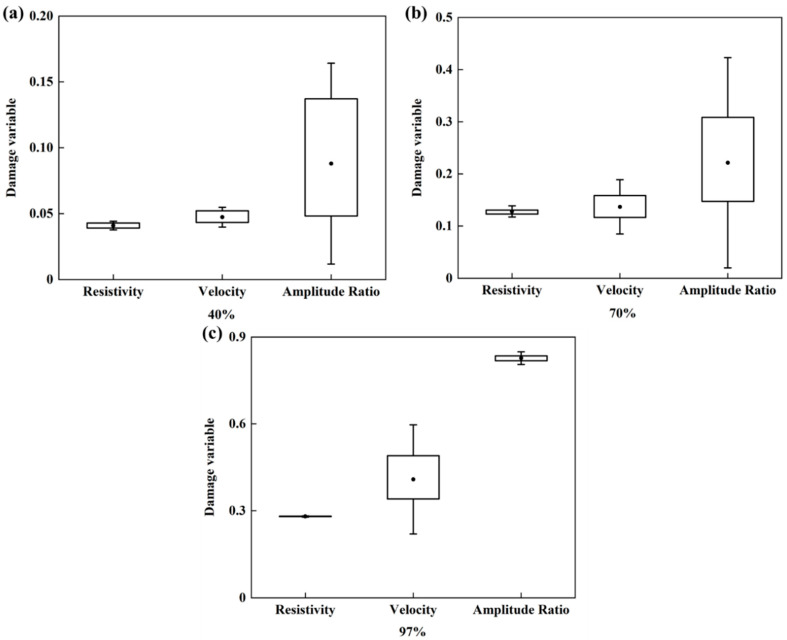
Stability of the damage variables derived from the NDT parameters. (**a**–**c**) correspond to three separate sandstone specimens for which the peak load reached 40%, 70% and 97% of the strength during cyclic loading, respectively.

The ultrasonic and EIS tests were implemented three times for each specimen, before and after damage. The spread of the three measurements for each parameter can be used to assess the stability of the NDT parameters. Three sandstone specimens that were subjected to different degrees of damage were selected, and three values for the damage variable were calculated from the three repeated measurements for the P-wave velocity, wave amplitude attenuation and resistivity. The results are plotted as a box line diagram in Figure 13.

The results show that damage variables derived from resistivity and amplitude attenuation are less stable when the stress level is low. As the damage accumulates, the stability of the damage variables derived from resistivity and amplitude attenuation increases. The damage variables that are derived from the wave amplitude attenuation and from the resistivity become similarly stable when a specimen is broken. However, the damage variable derived from the P-wave velocity becomes less stable as the level of damage increases, so that when the specimen fails, the damage variable derived from resistivity is the most stable, followed by that derived from wave amplitude attenuation, and the damage variable derived from the P-wave velocity is the least stable.

The reason for these different stabilities is that the three parameters rely on different mechanisms to detect damage. In sandstone, both resistivity and wave amplitude attenuation reflect damage detected via the propagation of electrical current and ultrasonic waves through the pore structure, while the P-wave velocity captures damage detected by the propagation of ultrasonic waves around pores as the waves follow the continuous medium. When the degree of damage is low, the specimen has fewer pores and so electrical current and ultrasonic waves do not propagate along a specific pore channel, which results in the derived damage variables being relatively unstable. Fewer pores means that the medium is more continuous for ultrasonic wave propagation, and so the damage variable derived from ultrasonic wave velocity is relatively stable. In contrast, when the degree of damage is high, continuous pore channels form inside the specimen and the damage variables derived from resistivity and amplitude attenuation are more stable. For high levels of damage, the proportion of space occupied by a continuous medium is reduced, so the damage variables derived from the ultrasonic wave velocity are less stable.

### 4.2. Mechanisms Behind Damage Measurements

Ultrasonic testing and EIS testing are NDT methods that measure damage by detecting changes in the internal structure of the specimen. According to the results in the previous section, the difference between the propagation mechanisms for ultrasonic waves and electrical current leads to a difference between the derived damage variables and between the stability of damage testing. Kassab believes that the P-wave velocity measurement can be interpreted as a measure of damage based on the propagation of ultrasonic waves around pores and thus primarily reflects the continuous medium of the rock [27]. On the other hand, Wang thought the resistivity measurement can be interpreted based on the propagation of current in the pore [28]. Both measures are associated with the porosity of the rock, but there are differences between the mechanisms that control the two measurements [29]. To investigate this, we established the relationships between P-wave velocity and porosity, and between resistivity and porosity, using theoretical and empirical models. Using porosity as a common factor, a relationship between P-wave velocity and resistivity was found, and validated using the results from our experiments.

Many models describe the relationship between P-wave velocity and rock porosity, including the Gassmann model, which is based on theoretical assumptions, and the Raymer model, which is based on empirical relationships. The Gassmann model is a theoretical model based on the close relationship between the P-wave velocity and the dynamic elastic modulus of rock [30]. The Gassmann formula for calculating the P-wave velocity is as follows:

(6) $$V_{p}^{2}\rho =\left(K_{d}+\frac{4}{3}G_{d}\right)+\frac{{\left(1-\frac{K_{d}}{K_{s}}\right)}^{2}}{\left(1-\varphi -\frac{K_{d}}{K_{s}}\right)\frac{1}{K_{s}}+\frac{\varphi }{K_{f}}}$$

where, *V_p_* is the P-wave velocity (m/s); *ρ* is the bulk density (kg/m^3^); *φ* is the porosity; *G_d_* is the shear modulus for rock (GPa); *K_d_*, *K_s_*, and *K_f_* are the moduli for rock, solid fraction of rock, and liquid fraction of rock (i.e., pore fluid), respectively. *K_d_* and *G_d_* are calculated using Krief’s formula, which includes the elastic and shear moduli for the rock solid fraction, *K_s_* and *G_s_*, respectively, the porosity φ, and the Krief index, *κ* (*κ* = 3) [31]. Check Table 1 for the values of the above parameters.

(7) $$K_{d}=K_{s}{\left(1-\varphi \right)}^{\kappa /\left(1-\varphi \right)}$$

(8) $$G_{d}=G_{s}{\left(1-\varphi \right)}^{\kappa /\left(1-\varphi \right)}$$

Raymer’s formula is an empirical formula for calculating acoustic velocity for materials with different porosities, based on measured acoustic logging data [32]. The formula is calculated as follows, where *v_s_* and *v_f_* are the P-wave velocities for the rock solid fraction and pore fluid, respectively:

(9) $$v_{p}={\left(1-\varphi \right)}^{2}v_{s}+\varphi v_{f}$$

**Figure 14:**
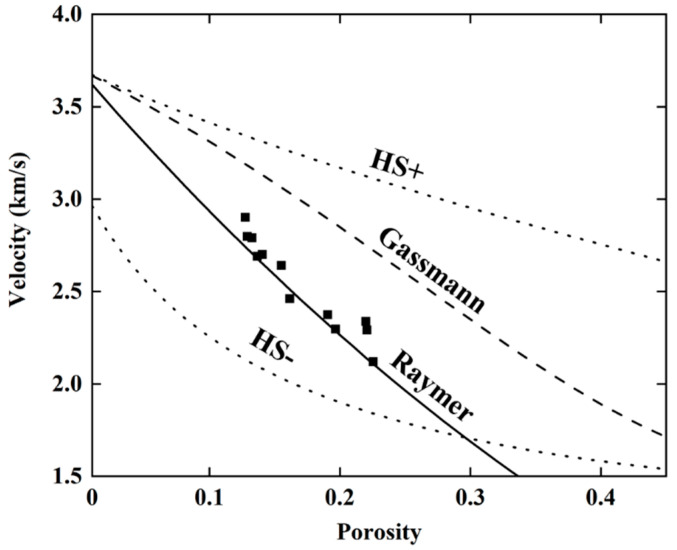
Different P-wave velocity-to-porosity models.

The Hashin-Shtrikman (HS) bounds are the theoretical upper and lower boundaries for the elastic and shear moduli and can therefore be used to calculate upper and lower boundaries for the P-wave velocity for rocks [33]. Figure 14 shows the relationship between the P-wave velocity and porosity, calculated using the above models for porosities ranging from 0% to 45%, with a scatterplot of the experimental data superimposed, where porosities are measured separately for all 12 specimens after damage. The model results generally fall within the HS boundaries, and the P-wave velocity calculated by the models decreases with the increase in porosity. The measured data are in good agreement with the Raymer model based on empirical relationships, which is consistent with the results in the previous literature [34].

The relationship between resistivity and porosity can be described by a self-similar model, which is based on self-consistent effective medium theory, or by the Archie model, which is based on empirical relationships. In the self-similar model, resistivity is continuously iterated and its relationship with porosity is then given by the following expression [35]:

(10) $$\rho ={\left(\frac{\rho -\rho_{s}}{\rho_{s}-\rho_{f}}\right)}^{m}\rho_{f}\varphi^{-m}$$

where *ρ*, *ρ_s_* and *ρ_f_* are the resistivity (Ω.m) of the rock, matrix and pore solution, respectively; *φ* is the porosity; and *m* is the cementation exponent, which equals 2.2 for the moderately cemented sandstone specimens in this study [14].

The Archie model is an empirical equation based on measured resistivity logging data [36]. The equation for the Archie model assumes that the resistivities for pure and saturated sandstone are proportional to the resistivity for the pore solution and includes tortuosity factor, *a*, which is equal to 0.8:

(11) $$\rho =a\rho_{f}\varphi^{-m}$$

**Figure 15:**
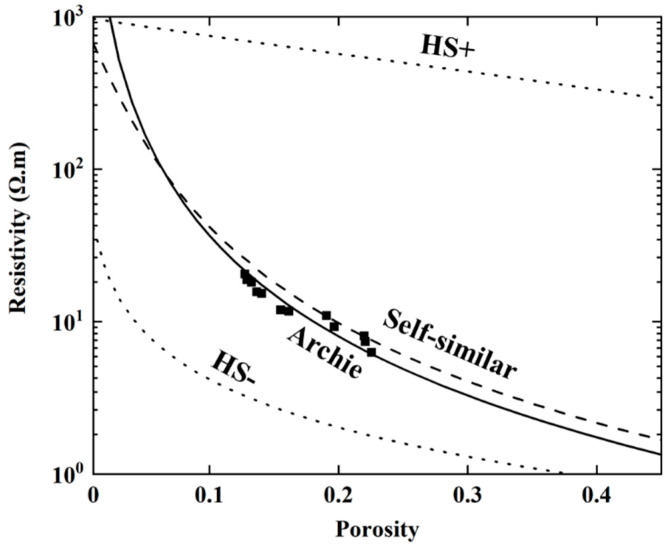
Different resistivity-to-porosity models.

If the effect of particle geometry is assumed negligible, then the HS boundary can also provide upper and lower boundaries for the resistivity [37]. Figure 15 shows the experimental data alongside the relationships between resistivity and porosity calculated using the different models. The results show that the resistivity of the sandstone decreases with increasing porosity and the experimental data are in good agreement with the Archie model [30]. When the porosity is low, the experimental data are distributed near the Archie model curve. When porosity is high, the experimental data are scattered between the curves for the Archie model and for the self-similar model.

**Figure 16:**
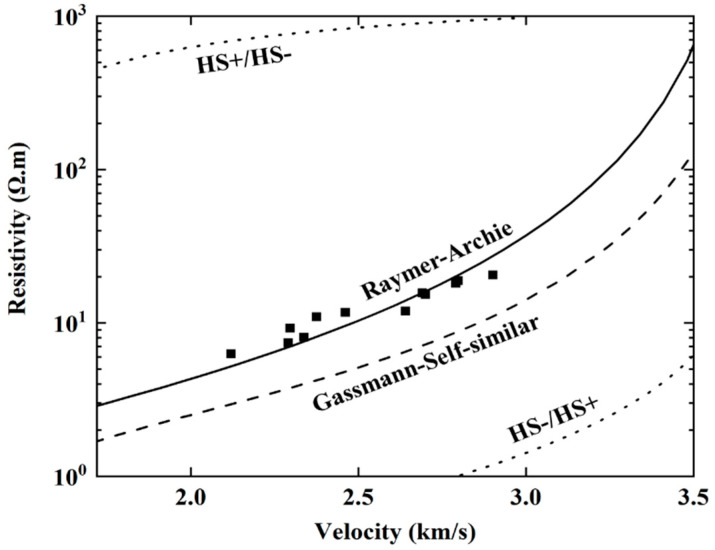
Different models of the relationship between the P-wave velocity and resistivity.

The relationships between the P-wave velocity and porosity with the relationships between resistivity and porosity were combined to establish the relationship between the P-wave velocity and resistivity, using the porosity as a common variable. Figure 16 shows that the experimental data were generally consistent with the Raymer-Archie model, which was constructed from empirical data. This demonstrates the accuracy of the test data and proves that the differences between damage measured parameters of sandstone is caused by the differences in reflecting changes to pore condition between damage measuring methods.

## 5. Conclusions

Ultrasonic tests, EIS tests and CT scan tests were carried out on sandstone specimens before and after subjecting the specimens to different levels of damage, and the unloading elastic moduli were obtained from the resulting stress-strain curves. Four NDT parameters: P-wave velocity, first wave amplitude attenuation, resistivity, and CT-based effective bearing area, and one destructive test parameter, the elastic modulus, were compared in terms of derived damage variables for sandstone.

The results show that the elastic modulus result in damage variables that agree closely with those derived from NDT damage measurement parameters, justifying the use of NDT test parameters to determine sandstone damage variables. The P-wave velocity agrees more closely than the wave amplitude attenuation with the other damage measurement parameters but is only moderately stable. The wave amplitude attenuation is more sensitive than the other measurement parameters to sandstone damage, and its stability increases significantly as the level of damage increases. Damage variables calculated from the CT scan tests capture microscopic properties, but the finite resolution of the CT scans means that the derived damage variables have lower values than those derived from macroscopic measurement methods.

The resistivity and wave amplitude attenuation reflect the condition of the pore structure, while the P-wave velocity reflects the condition of the continuous medium inside the sandstone. The relationship between the P-wave velocity and resistivity was modeled by considering the relationship of both to porosity. The experimental data were consistent with the Raymer-Archie model, verifying that the different responses of the measured parameters to porosity is what drives the differences between the different damage measurement parameters.

## Figures and Tables

**Table 1:** Values used for the material parameters in Equations (6)–(11) (see text for units).

*ρ_s_*	*ρ_f_*	*K_s_*	*K_f_*	*G_s_*	*v_s_*	*v_f_*
1000	0.29	25	2.25	8	3.7	1.4
